# A Scalable Spin‐Coating Strategy for Mesoporous Gold Thin Films toward miRNA Detection

**DOI:** 10.1002/advs.77604

**Published:** 2026-10-08

**Authors:** Aditya Ashok, Emtiaz Ahmed, Arya Vasanth, Mandy Hei Man Leung, Miharu Eguchi, Md Shahriar A. Hossain, Mostafa Kamal Masud, Carlos Salomon, Yusuke Yamauchi

**Affiliations:** ^1^ Australian Institute for Bioengineering and Nanotechnology The University of Queensland Brisbane Queensland Australia; ^2^ Department of Materials Process Engineering, Graduate School of Engineering Nagoya University Chikusa‐ku Nagoya Japan; ^3^ Institute for Advanced Research (IAR) Nagoya University Nagoya, Aichi Japan; ^4^ Faculty of Science and Engineering Waseda University Shinjuku Tokyo Japan; ^5^ School of Mechanical and Mining Engineering, Faculty of Engineering, Architecture and Information Technology (EAIT) The University of Queensland Brisbane Queensland Australia; ^6^ Translational Extracellular Vesicles in Obstetrics and Gynae‐Oncology Group, UQ Centre for Clinical Research (UQCCR), Faculty of Medicine, and UQ Centre for Extracellular Vesicle Nanomedicine The University of Queensland Brisbane Queensland Australia; ^7^ Department of Convergent Biotechnology and Advanced Materials Science Kyung Hee University, Giheung‐gu Yongin‐si Gyeonggi‐do South Korea

**Keywords:** biosensors, electrochemical sensing, electroless deposition, mesoporous gold, miRNA, spin coating, thin films

## Abstract

Mesoporous gold (mAu) films promise biosensing and electrochemical sensing due to their electroactive surface area, conductivity, and biocompatibility. However, conventional fabrication approaches, including electrodeposition and templating, often require substrate‐specific processing and can be challenging to scale, limiting the broader integration of mAu films into practical sensing platforms. Here, we report scalable spin‐coating of mAu films on transparent substrates with tunable thickness and uniform mesoporous framework. Electrochemical analyses reveal that additional coating cycles progressively increase the electrochemically active surface area and reduce the interfacial charge‐transfer resistance, yielding a corresponding rise in the differential‐pulse voltammetric signal, consistent with a mesoporous architecture that promotes efficient redox access and analyte adsorption. To demonstrate biosensing capability, a label‐free electrochemical assay for the ovarian cancer‐associated microRNA (miRNA) hsa‐miR‐200c‐3p as a model target is developed. The sensor responds monotonically from 1 aM to 10 pM, with a linear calibration (R^2^ = 0.9831), an LOD of 1.72 aM, and an LOQ of 2.98 aM without amplification. This study establishes spin‐coating as a scalable approach to mesoporous Au electrodes for ultrasensitive nucleic acid biosensing. By decoupling mesoporous‐metal fabrication from substrate conductivity, it enables high‐surface‐area electrodes on transparent, flexible, insulating supports and extension to RNA biomarkers for miniaturized point‐of‐care diagnostics; uniformity and long‐term stability remain challenges.

## Graphical abstract

A scalable spin‐coating strategy produces uniform mesoporous Au thin films with tunable thickness and high electroactive surface area on transparent substrates. The resulting architecture enables label‐free, amplification‐free detection of ovarian cancer‐associated miRNA hsa‐miR‐200c‐3p from 1 aM to 10 pM, achieving a 1.72 aM detection limit with high selectivity and reproducibility.

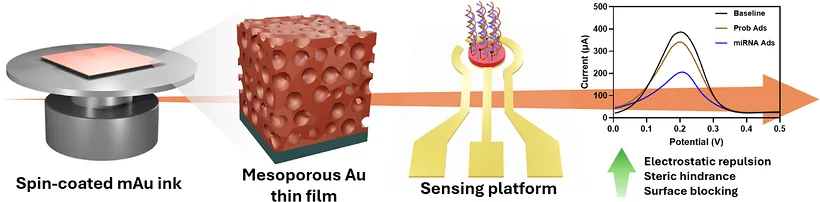

## 1 Introduction

Mesostructured noble‐metal thin films are an appealing materials platform because they combine intrinsically high electrical conductivity, chemical stability, and process‐compatibility with nanoscale architecture control. Noble metals such as gold (Au), silver (Ag), platinum (Pt), and their alloys remain among the most widely used functional transducer materials due to facile surface chemistry (notably thiol–metal bonding), long‐term stability under aqueous/physiological conditions, and reliable charge transport [1, 2, 3, 4]. While semiconducting nanomaterials provide cost‐effective and photoactive alternatives for biomolecular sensing [5], the broader technological value of noble metals extends beyond any single application: creating continuous, robust, and scalable mesoporous noble‐metal coatings on practical substrates would enable integration into miniaturized, multifunctional, and mechanically compliant devices where high surface area and rapid mass/charge transport are required [6].

Mesoporous materials have traditionally been produced through templating and non‐templating routes. Soft‐templating, in which surfactants or block copolymers self‐assemble into micelles that guide the formation of frameworks, is widely used for mesoporous oxides, carbons, and metals [7, 8, 9]. Block copolymer‐directed self‐assembly, pioneered by Wiesner and co‐workers, enables fine control over pore size, symmetry, and connectivity and can generate complex three‐dimensional architectures, such as gyroids [10, 11, 12]. Despite this structural precision, such approaches often require tightly tuned polymer–inorganic interactions, complex precursor formulations, and narrow processing windows to achieve long‐range order and reproducibility [13, 14]. Template removal can demand high‐temperature calcination or harsh chemical treatments that risk framework collapse or contamination, and scaling to large‐area thin films or coating onto diverse substrates remains challenging [15, 16]. Hard‐templating (nanocasting) using rigid scaffolds (e.g., SBA‐15, KIT‐6) offers high structural fidelity, but infiltration and template removal complicate scalability [10]. Template‐free strategies, such as dealloying, gas evolution, and phase separation [17], can generate hierarchical porosity and multimodal pore‐size distributions favorable for mass transport, yet controlling pore ordering, film adhesion, and uniformity in thin‐film formats can be difficult [16]. In the specific case of mesoporous Au thin films, micelle‐assisted electrochemical deposition is a standard route that delivers uniform porosity and high electroactive surface area [17], but it inherently requires conductive substrates and precise electrochemical control. Solution‐processing methods such as evaporation‐induced self‐assembly (EISA) have been powerful for oxides and hybrid films [15, 16], and dealloying of AuCu alloys yields nanoporous metals [18, 19]. More recently, liquid‐metal‐templated routes have produced mesoporous noble‐metal thin films and micropatterns with enlarged electroactive surface area and improved antifouling behavior for biosensing [20]. However, a general and scalable strategy that enables mesoporous metal coatings on arbitrary substrates, including non‐conductive substrates, without relying on electrochemical processes remains unavailable. Closing this gap is essential for translating mesoporous metallic architectures into practical device components. Mesoporous gold thin films are particularly attractive for electrochemical biosensing. Their interconnected pore network provides a large electrochemically active surface area and a high density of accessible sites that amplify faradaic signals, while short intra‐pore diffusion paths and continuous metallic connectivity sustain rapid mass and charge transport. Gold further offers facile and selective thiol–Au surface chemistry for oriented probe immobilization, together with chemical stability and biocompatibility under physiological conditions. Together, these features make mesoporous Au well suited to label‐free, amplification‐free electrochemical readouts, in which signal transduction relies on interfacial blocking upon target capture [20].

Here, we establish a scalable, versatile method for synthesising mesoporous gold (mAu) thin films by spin‐coating onto indium tin oxide (ITO) glass [21]. The film thickness is readily tuned by the number of coating cycles while preserving uniform mesoporosity across the coated area. This solution‐processable route extends mesoporous noble‐metal coatings beyond conventional electrodeposition‐limited conductive supports and provides a practical pathway to integrate mesoporous metals with transparent, biocompatible, and device‐relevant substrates for large‐area optoelectronic and sensing platforms [22, 23, 24]. The resulting interconnected pore network provides a high density of accessible surface sites and short diffusion pathways, while maintaining continuous metallic conductivity, which is broadly beneficial for interfacial processes that demand efficient mass transport and electron transfer [25, 26, 27].

As a proof‐of‐concept, we demonstrate the utility of these spin‐coated mAu films in electrochemical nucleic acid detection, focusing on the ovarian cancer–associated miRNA hsa‐miR‐200c‐3p. The miRNAs are attractive biomarkers because they exhibit disease‐associated dysregulation, pathway specificity, and stability in biofluids [28, 29], yet their analysis is technically demanding owing to extremely low abundance (attomolar to femtomolar), short sequence length (∼22 nt) [30], and high sequence homology within families, which requires single‐nucleotide discrimination under complex matrix conditions [31, 32]. Conventional workflows such as RT‐qPCR/digital PCR, microarrays, and next‐generation sequencing provide sensitivity and breadth but typically require enzymes, labeling, multistep sample preparation, specialized infrastructure, and extended turnaround times [33, 34]. These limitations motivate the development of amplification‐free, rapid electrochemical strategies capable of single‐step hybridization readouts on manufacturable substrates [34, 35].

In this study, using the spin‐coated mAu films, we developed a label‐free, amplification‐free electrochemical assay with a turnaround time of <1.5 h, employing a three‐stage measurement workflow and a quantitative readout defined as hybridization‐induced absolute adsorption (%iR_a_). The optimized sensor exhibited a concentration‐dependent increase in %iR_a_ from 1 aM to 10 pM with a linear working region (R^2^ = 0.9831), achieving a limit of detection of 1.72 aM and a limit of quantification of 2.98 aM. Replicate measurements yielded a relative standard deviation below 10%, and nonspecific controls, including scrambled sequences, triple‐base‐mismatched sequences, and nonspecific miRNAs, produced negligible signals, confirming robust sequence selectivity. Collectively, this work positions spin‐coated mesoporous noble‐metal films as a substrate‐flexible, scalable synthetic platform, with biosensing presented here as one representative application enabled by the accessible mesoscale architecture and continuous metallic conduction.

## 2 Results and Discussion

The mAu thin films were fabricated through a solution‐processed route involving the preparation of a polymer‐stabilized mAu ink, followed by spin‐coating onto ITO‐coated glass substrates, as shown in Figure 1. The spin‐coating route developed here does not require a conductive substrate, since film formation proceeds through controlled solvent evaporation and micelle self‐assembly rather than electrochemical nucleation; it is therefore compatible with insulating and transparent supports. For the electrochemical biosensing demonstration, an electrically addressable electrode is required, so conductive ITO‐coated glass (sheet resistance ≈ 8–12 Ω·sq^−^^1^) was used. ITO additionally offers a mechanically robust, scratch‐resistant contact that maintains electrical continuity under repeated alligator‐clip connections during successive electrochemical measurements. The diblock copolymer polystyrene (18 kDa)‐blocked‐polyethylene oxide (7.5 kDa) (PS_18k_‐*b*‐PEO_7.5k_) was selected because its relatively large hydrophobic PS_18k_ block templates correspondingly large, well‐connected mesopores that favor analyte diffusion and probe accessibility and yield a simpler, more open pore network. This molecular weight offers a favorable balance between accessible surface area and framework robustness, which we found well suited to sensitive electrochemical nucleic‐acid detection. In this approach, the PS_18k_‐*b*‐PEO_7.5k_ (Figure 1a), acting as a polymeric micelle, was employed to disperse the gold precursors and guide the formation of mesostructured Au domains through a self‐assembly process within the ink, as shown in Figure 1b. The oxygen plasma‐treated ITO‐glass substrates helped the ink spread uniformly across the substrate during spin coating, and controlled evaporation of the solvent induced the assembly of the micelle–Au framework into a porous architecture, as shown in the illustration (Figure 1c). The process began by dropping 20 µL of the prepared mAu ink, as shown in Figure 1 (i), on the fresh O_2_ plasma‐treated surface. Then, the substrate was spun at 600 rpm for 60 s to achieve uniform surface coverage (Figure 1 (ii)). The hydrophilic PEO block forms the micelle corona that anchors to the hydroxyl (‐OH) terminated ITO surface and promotes uniform ink spread, as a result of O_2_ plasma exposure. Finally, the spin‐coated electrodes were annealed (Figure 1 (iii)). Repeated coating cycle (CC) enabled systematic tuning of the film thickness while preserving the mesoporous nanostructure. Finally, the spin‐coated samples were thermally annealed and subjected to chemical treatment (i.e., THF) to remove the organic components (di‐block copolymer), yielding adherent mAu films with interconnected nanopores and a high surface area. Thus, this method provides a simple yet versatile alternative to electrochemical deposition, enabling the direct integration of uniform mesoporous Au films onto insulating or transparent‐conductive substrates.

**FIGURE 1:**
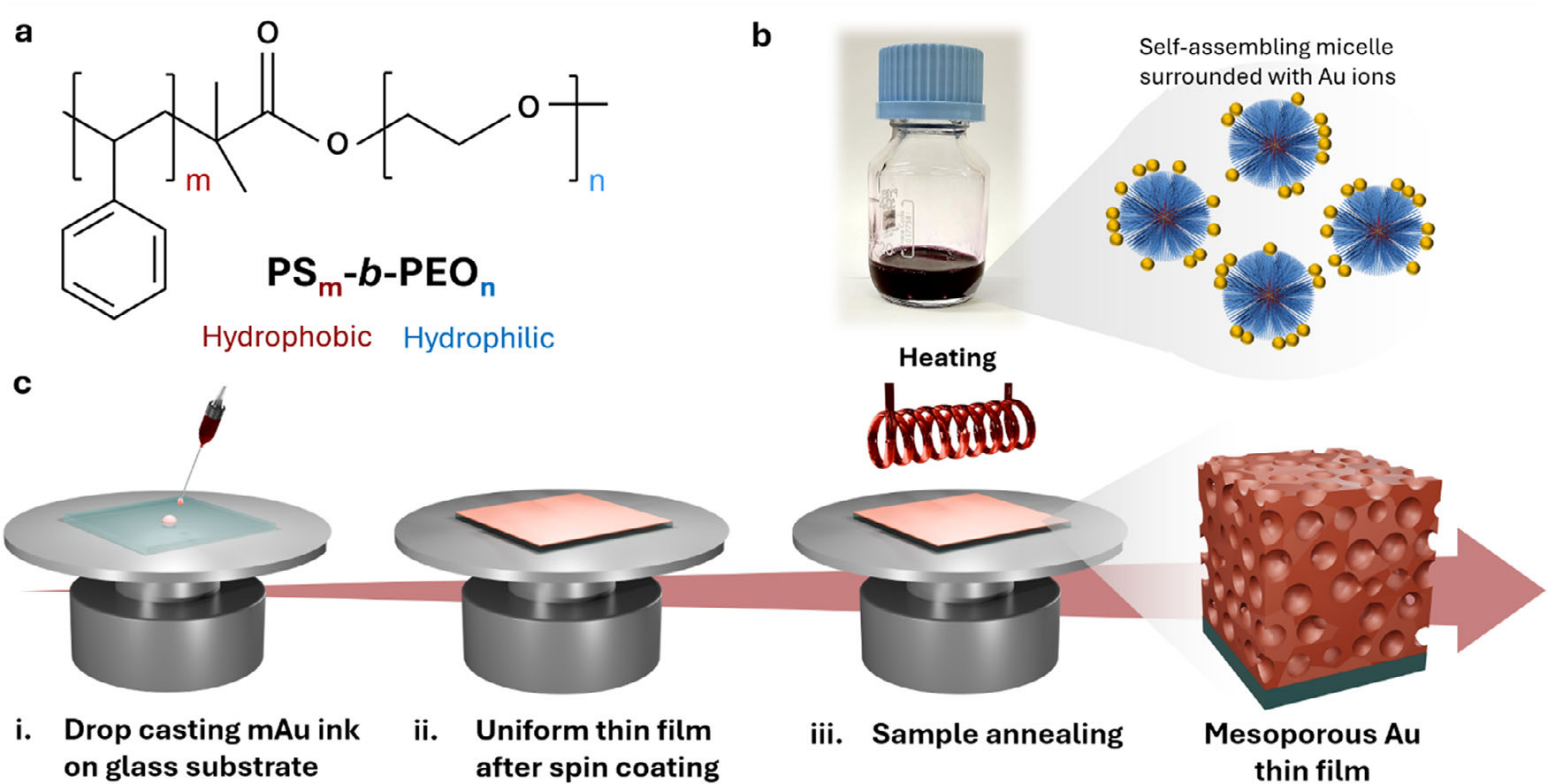
Illustration representing spin coating mAu. (a) Hydrophobic polystyrene_m_ blocked with hydrophilic poly(ethylene oxide)_n_ diblock copolymer (PS_18k_‐*b*‐PEO_7.5k_) and (b) mAu ink containing self‐assembling micelles. (c) Process involved in spin‐coating a mAu thin film.

The surface morphology and mesostructural features of the spin‐coated mAu films are investigated using field‐emission scanning electron microscopy (FESEM). As shown in Figure 2a, the films exhibit a continuous crack‐free architecture with uniformly distributed mesopores interconnected through a sponge‐like framework from 3 CC and above (supplementary Figure S1). The pore and framework sizes were quantified from high‐resolution FESEM images, revealing systematic variation with the number of spin‐coating cycles (Figure 2b,c). For films obtained from a single cycle, the pore diameters were relatively small, averaging 17.4 nm, and the frameworks were thinner at 21.6 nm, indicating a lower material loading per deposition. With increasing CCs, the framework thickness progressively increased at ca. 181.23 nm per CC (Figure 2d), gradually increasing the average pore size and thinner ligaments while preserving the overall mesoporous structure. This trend highlights that repeated spin‐coating controls the overall film thickness and modulates the nanoscale architecture, enabling fine‐tuning of the surface area and structural robustness of the mAu films in annealed electrodes. In principle, film thickness can also be reduced by increasing the spin speed; here, however, the usable speed window is limited by mesostructure integrity. Speeds above the optimized 600 rpm collapse the soft‐template micelles into a non‐porous film, whereas lower speeds cause pronounced edge‐bleeding. We therefore fixed the spin speed at 600 rpm and controlled film thickness through the number of CCs, which preserves the mesoporous framework while systematically varying thickness. Tunability of pore and framework dimensions is critical for optimising surface reactivity and probe loading in biosensing applications. The quality of the mAu films is examined using energy‐dispersive X‐ray spectroscopy (EDX). The spectrum shown in Figure S2 for the 3 and 5 CC electrodes indicates the presence of only Au, with an atomic percentage (At.%) of approximately 78%, followed by carbon and oxygen.

**FIGURE 2:**
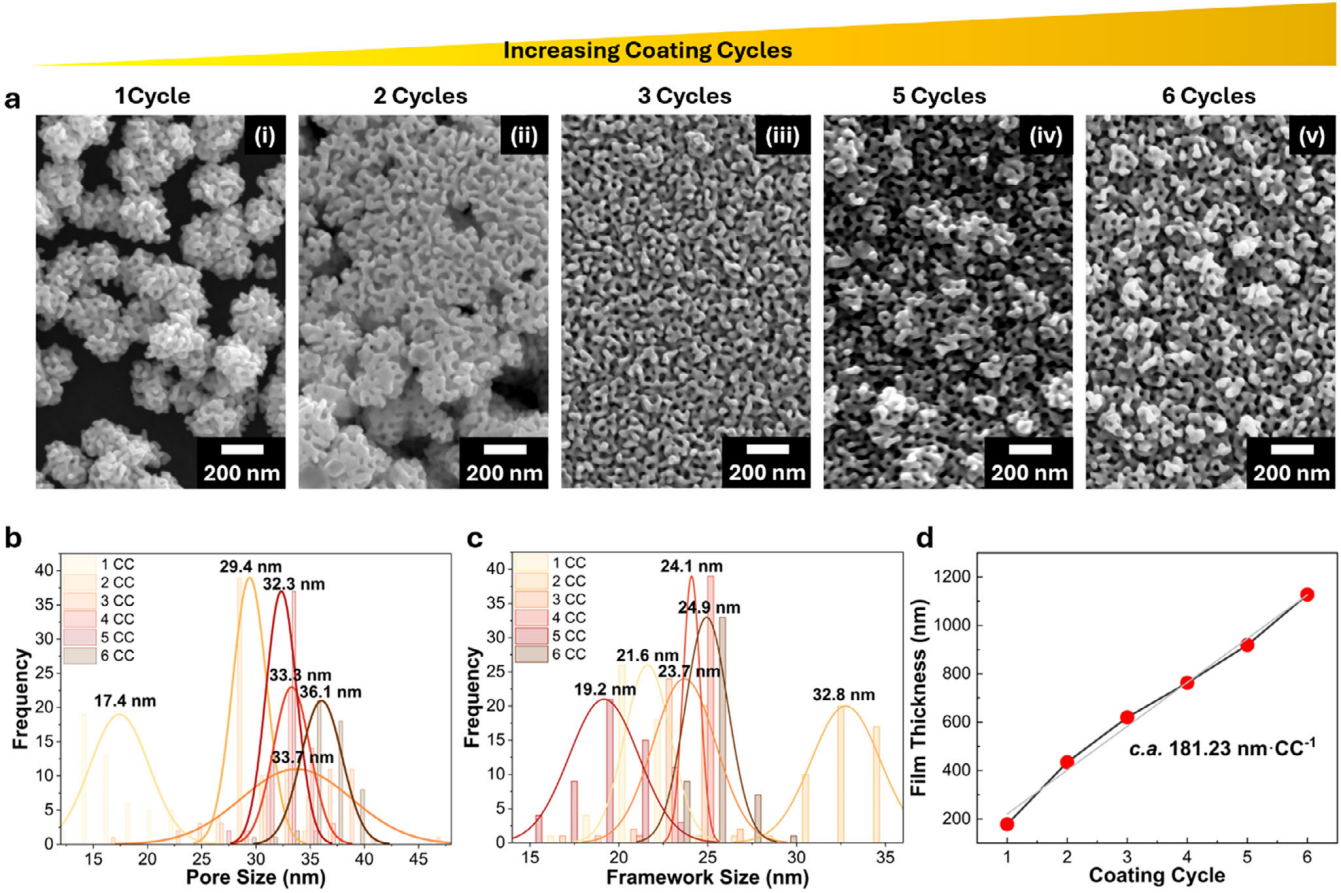
Structural information at different CC. (a) Mesoporous morphology of Au at (*i‐v*) increasing CC as indicated above the images, respectively. (b) Pore size distribution, (c) framework size, and (d) average film thickness measured across increasing CC.

Scanning transmission electron microscopy (STEM) analysis was performed on a cross‐sectional lamella prepared by focused ion beam (FIB) milling, enabling detailed examination of the nanoarchitectured thin film. As shown in Figure 3a, a randomly distributed mesoporous network extends continuously from the substrate to the surface of the 1 CC mAu film, with an overall film thickness of approximately 264 nm. At higher magnification in the region indicated by the red box in Figure 3a, well‐defined interfacial layers are revealed. Figure 3b, identifies four distinct regions across the cross‐section. From left to right, region (i) corresponds to the glass substrate, followed by 12 nm thick ITO as region (ii) in green, then a thin region (iii) of approximately 6 nm, indicated in violet, is the oxygen‐rich layer formed as a result of oxygen plasma treatment, which promotes uniform spreading of the ink during spin coating. Finally, region (iv), highlighted in yellow, corresponds to the mesoporous Au (mAu) film. These observations are further corroborated by TEM elemental mapping presented in Figure S3. Crystallographic analysis obtained from X‐ray diffraction (XRD) confirms the dominant presence of Au with a preferred (111) orientation (Figure 3c), along with minor peaks corresponding to the underlying ITO layer.

**FIGURE 3:**
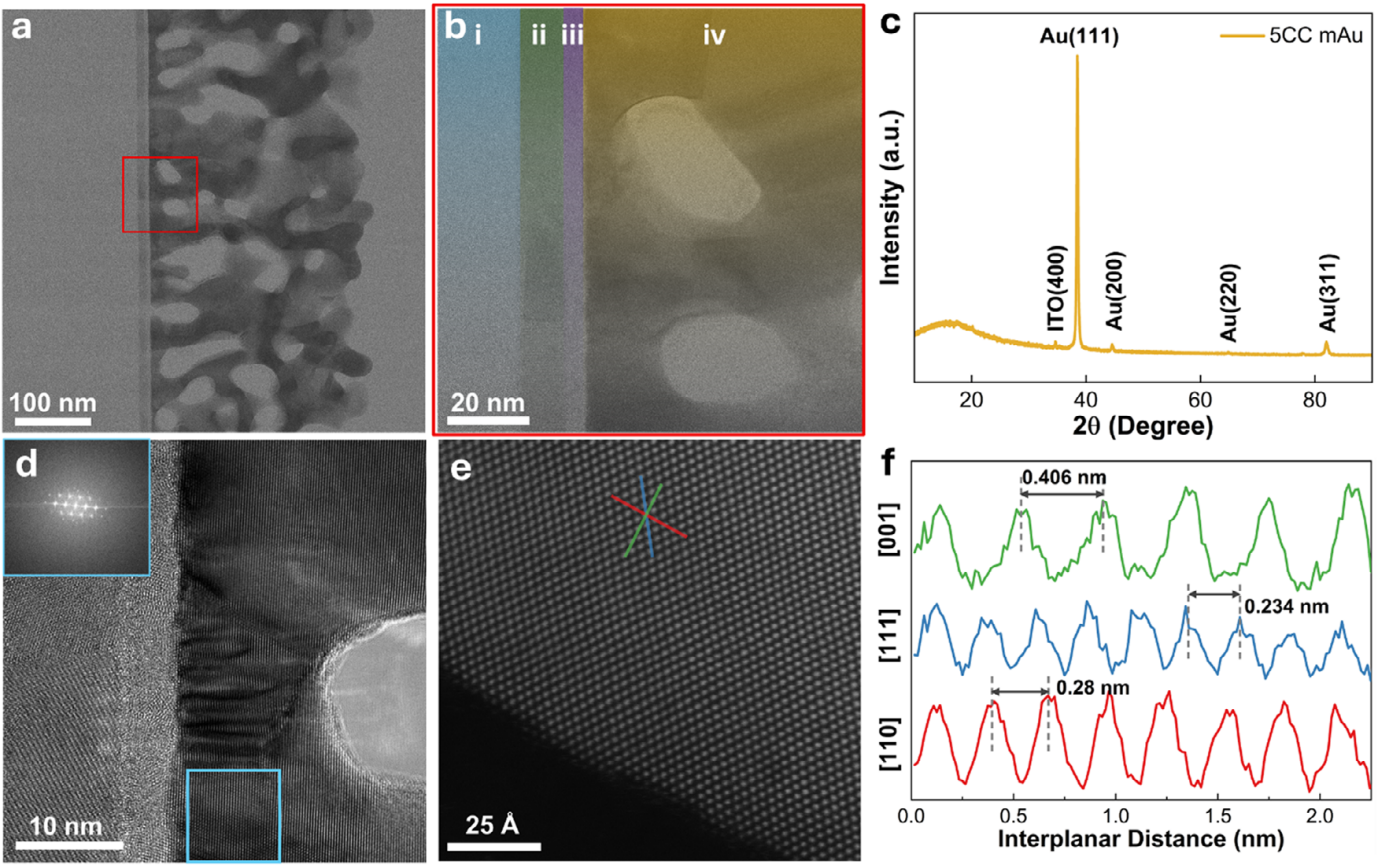
S/TEM and XRD of spin‐coated mAu. (a) Bright field STEM cross‐section of 1 CC mAu at 100 nm scale. (b) STEM image along the substrate‐film interface with four different regions indicated in false color. (c) XRD of 5 CC mAu film. (d) HR‐TEM image with lattice structures at the ITO/mAu interface. (Inset: FFT) (e) Dark field HR‐STEM image of local atom arrangement along the pore edge (scale bar: 25 Å) and (f) their corresponding d‐spacing extracted along the planes [001] in green, [111] in blue and [110] in red, respectively.

High‐resolution transmission electron microscopy (HR‐TEM) images in Figure 3d provide a lattice‐resolved view of the cross‐sectional ITO/mAu interface. An approximately 6 nm‐thick amorphous ITO layer is observed at this interface, attributed to high‐power O_2_ plasma treatment and expected to enhance surface wettability for effective spin coating. This amorphous, oxygen‐rich layer is a thin surface region generated by the high‐power O_2_‐plasma treatment; it sits above the underlying ∼12 nm crystalline, conductive ITO that provides the electrical pathway required for electrochemical readout. The HR‐TEM image further reveals a highly directional arrangement of the mAu, indicative of effective post‐annealing. This observation is supported by the diffraction patterns (Figure 3d, inset) obtained via fast Fourier transform (FFT) from the region highlighted by the blue box. Additional HR‐TEM images in Figure S4, collected from the mid‐section of the sample and along pore edges, consistently confirm a highly directional atomic arrangement rather than the multi‐crystalline facets commonly observed in nanostructured thin films. However, lattice dislocation is evident, particularly extending from the pore edges toward the bulk of the film (Figure S4b,c). A curtaining artefact resulting from ion milling during focused ion beam (FIB) lamella preparation is visible on the sample surface (Figure 3d), and redeposition of the Pt protective layer is observed in Figure S4b. Figure 3e presents dark‐field HR‐STEM imaging, clearly resolving the lattice of mAu. The interplanar spacings along the [001], [111], and [110] directions are indicated by the green, blue, and red lines in Figure 3e, respectively. Extracted d‐spacing profiles yield values of 4.06 Å for d_001_, 2.34 Å for d_111_, and 2.8 Å for d_110_, as shown in Figure 3f, demonstrating that the mesoporous nanostructured thin film by spin‐coating.

To further demonstrate the influence of successive spin‐coating cycles, six mAu electrodes were arranged linearly and photographed against a printed background. As the number of CC increased, the films exhibited a progressively darker appearance, with reduced optical transmittance evident from the gradual obscuring of the underlying text (Figure 4a), consistent with increasing film thickness and Au loading. This visual trend was corroborated by electrochemical characterization. The electrochemically active surface area (ECSA) as shown in Figure 4b, determined by the oxygen stripping/redox technique, increased systematically with CCs, reflecting a higher density of mAu frameworks accessible for charge transfer. Similarly, electrochemical impedance spectra (EIS) revealed a decrease in charge‐transfer resistance with increasing film thickness, indicating improved electrical connectivity within the porous mAu network (Figure 4c). Additionally, the 6 CC electrode exhibited an impedance of just 6.39 Ω measured at 1 kHz frequency, while the 5.15 Ω of the electrode with only 1 CC at a similar frequency, as shown in the inset of Figure 4c. Moreover, differential pulse voltammetry (DPV) measurements showed an enhanced signal response with increasing CC, as shown in Figure 4d, confirming that the enlarged electroactive area directly translates into superior redox sensitivity. Overall, from Figure 4e, it is evident that the ECSA and its related DPV characteristics of the spin‐coated mAu electrodes increase linearly with increasing CCs. Together, these optical and electrochemical results validate that spin‐coating cycles are an effective approach for tuning the structural and functional performance of mAu film, while the measured impedance magnitude exhibited only modest CC‐dependent changes.

**FIGURE 4:**
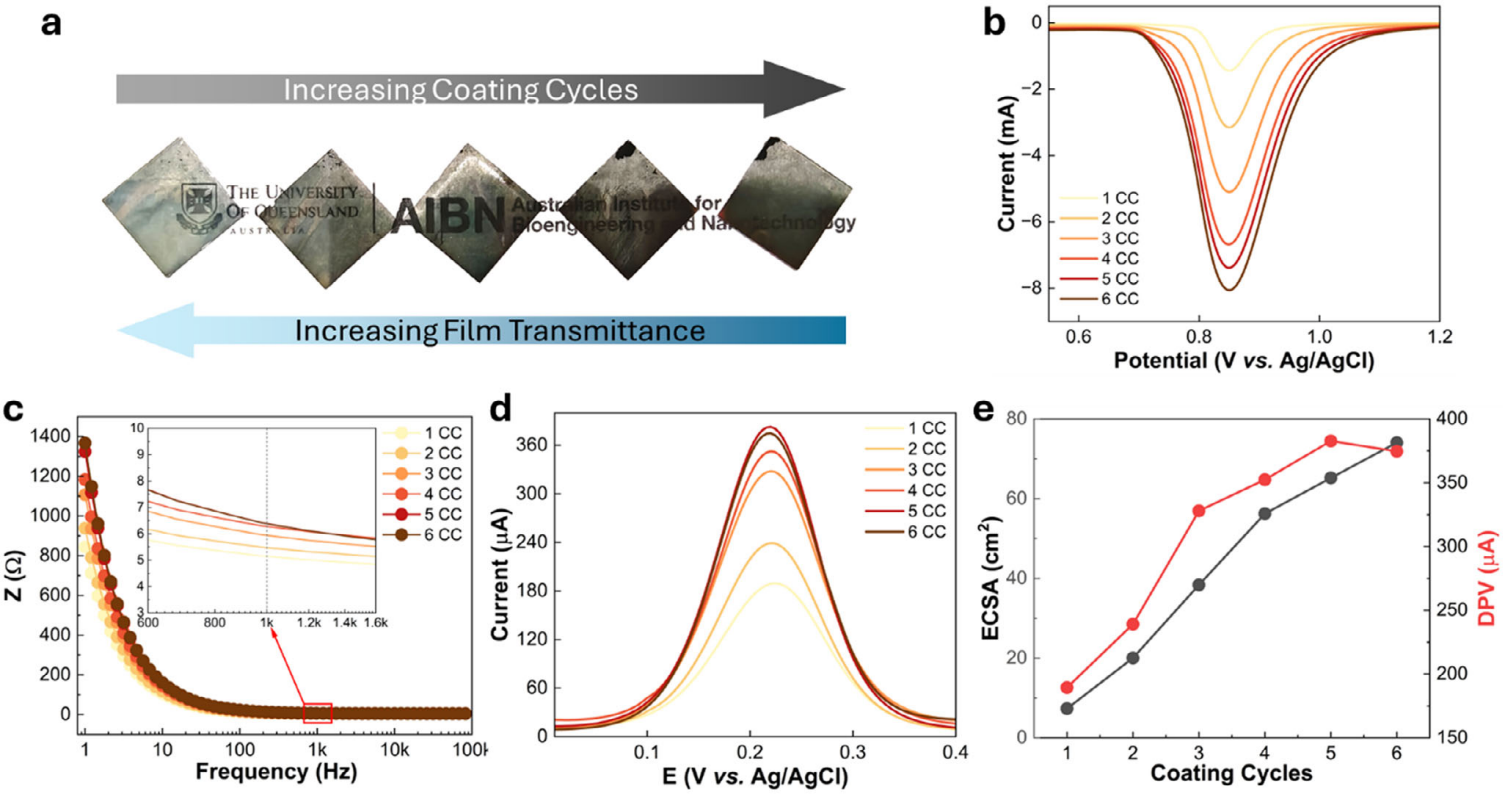
Sample micrograph and electrochemical characterization. (a) mAu film with increasing CC indicating a visual change in the light transmittance. (b) Surface area extracted from samples with increasing CC electrochemically (scan rates: 50 mV·s^−1^). (c) Impedance (Z) spectra obtained at different frequencies in 1× PBS, with inset indicating Z at 1 kHz, and (d) DPV performed in electrolyte containing 2.5 mM [K_3_Fe(CN)_6_] and 2.5 mM [K_4_Fe(CN)_6_] solution as analyte. (e) ECSA and DPV as a function of CC.

Following successful synthesis and functional characterization, we assessed the biosensing performance of novel mAu electrodes using an ovarian cancer‐associated miRNA as the target analyte, aiming to develop a highly sensitive miRNA sensor. Freshly fabricated electrodes were cleaned in two steps: (i) an ethanol rinse to remove any loosely adsorbed contaminants and (ii) electrochemical cleaning via consecutive cyclic voltammetry (CV) scans in 0.5 M H_2_SO_4_ solution. This oxidative treatment removed the residual organics and improved the electrochemical response of bare gold. Baseline behavior was then established by CV and DPV in the [Fe(CN)_6_]^3^^−^/^4^^−^ redox couple. Subsequently, reduced TCEP‐treated thiolated single‐stranded DNA (ssDNA) probes and 6‐mercapto‐1‐hexanol (MCH) were sequentially introduced onto the mAu electrode surface. The thiolated probes were functionalized with a short alkyl spacer at the 3′ end, allowing optimal orientation and reducing steric hindrance during target hybridization. Upon contact with the gold surface, the thiol groups undergo chemisorption, forming stable Au‐S bonds that provide strong anchoring of the specific capture probe to the mAu surface. MCH was employed as a blocking agent to passivate unmodified regions of the gold surface. Its terminal ‐SH group enables covalent attachment to free sites on the electrode, thereby preventing nonspecific adsorption of biomolecules during sensing. This backfilling step is essential for maintaining the sensor selectivity and surface integrity. After these modification steps, electrode behavior was re‐evaluated by CV and DPV in 2.5 mM [Fe(CN)_6_]^3^^−^/^4^^−^ to monitor changes associated with formation of the probe/MCH layer. The stability of the thiol‐functionalized sensing interface under the applied CV and DPV conditions is also investigated, as thiol‐based self‐assembled layers on Au can undergo potential‐ and time‐dependent structural rearrangement or desorption. Previous studies have shown that the electrochemical stability of thiol and disulfide monolayers on Au depends on factors including the applied potential, exposure time, molecular coordination, electrolyte environment, and Au surface structure [36]. To experimentally assess the stability of the probe‐functionalized mAu interface under the CV and DPV conditions used in this study, FTIR spectra were recorded for bare mAu and probe/MCH‐functionalized mAu electrodes before and after CV and DPV interrogations (Figure S5). Relative to bare mAu, the probe/MCH‐functionalized electrode exhibited substantially enhanced spectral features within the molecular fingerprint region, consistent with formation of the biomolecular surface layer. Importantly, the overall spectral profile of the functionalized interface was largely retained following CV and DPV measurements. Although modest differences in band intensity were observed between the pre‐ and post‐DPV spectra, no substantial loss of the characteristic spectral features of the probe/MCH‐modified interface was evident. These observations support retention of the thiolated ssDNA/MCH layer during the applied CV and DPV measurements and indicate that the probe‐functionalized mAu interface is sufficiently stable under the electrochemical conditions employed in this study.

Finally, the analytical sensing step was based on specific hybridization between the immobilized thiolated DNA probe and the mature form of hsa‐miR‐200c‐3p, a clinically relevant miRNA biomarker known to be overexpressed in ovarian cancer. Hybridization was performed by incubating modified electrodes with a set of synthetic miRNA samples (target and control) under controlled conditions. Post‐hybridization, the electrochemical response was again characterized using DPV and CV to assess the hybridization efficiency. A schematic overview of the workflow is shown in Figure 5a.

**FIGURE 5:**
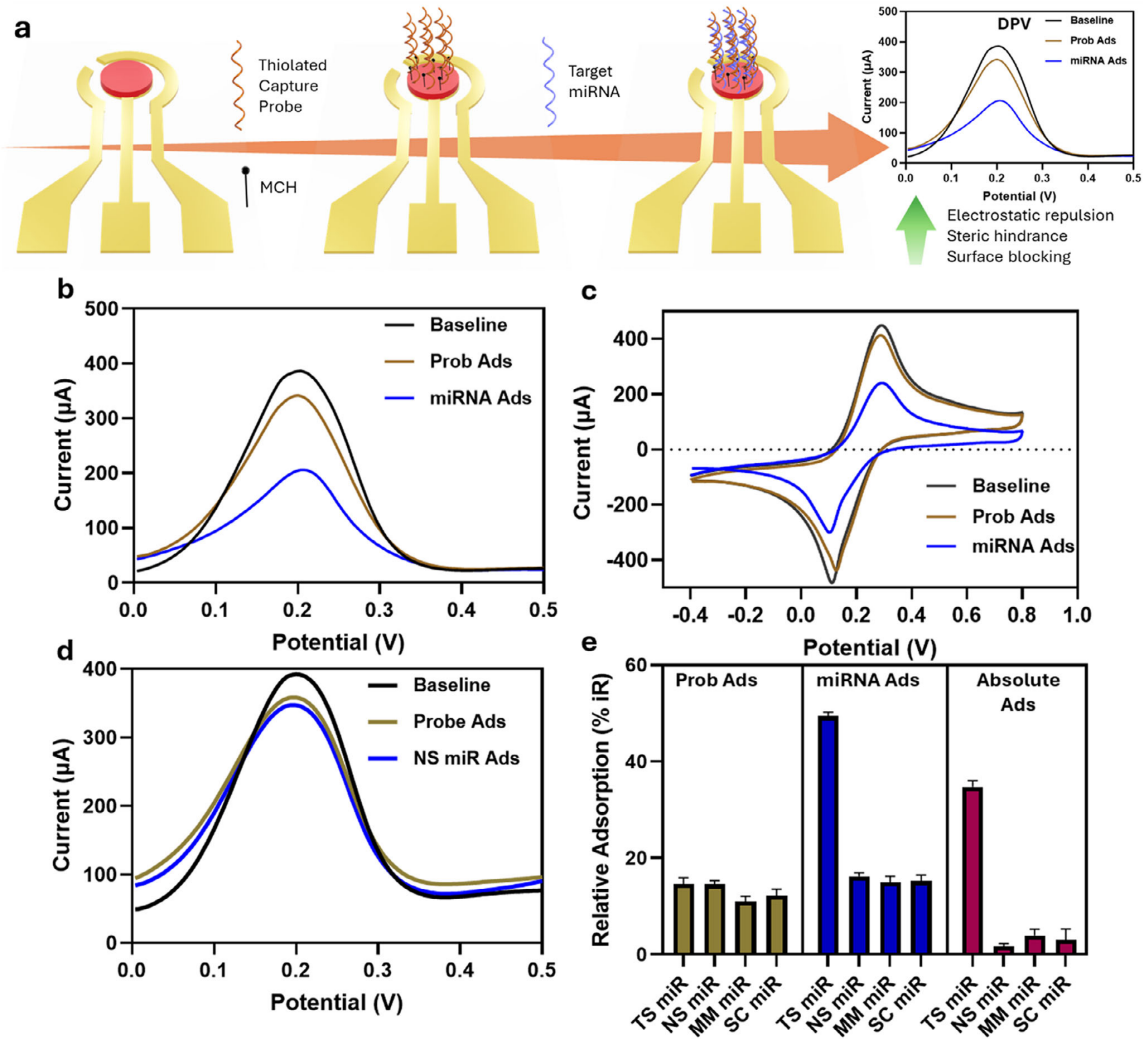
Schematic representation and electrochemical monitoring of the mAu‐based miRNA assay. (a) Schematic of the assay principle. (b) DPV traces for bare, probe+MCH, and post‐hybridization electrodes showing stepwise current suppression with surface modification and target capture. (c) CV corroboration: decreased anodic/cathodic currents and larger ΔE_p_ after probe+MCH assembly, with further suppression upon hybridization (scan rates: 50 mV·s^−1^). (d) Representative DPV response obtained with a non‐specific miRNA control. (e) Quantitative relative and absolute adsorption responses for target (TS), non‐specific (NS), mismatched (MM), and scrambled (SC) miRNAs, demonstrating sequence selectivity. Relative adsorption was calculated as the difference between %iR of miRNA hybridization and %iR of probe+MCH modification. (Error bars represent mean ± SD from n = 3 independently prepared electrodes).

To enable the sensitive and selective detection of the ovarian cancer–associated biomarker hsa‐miR‐200c‐3p, we optimized the mAu surface chemistry to form a passivated, well‐ordered interface that supports high‐affinity nucleic acid hybridization. Electrochemical readouts, such as DPV and CV in the [Fe(CN)_6​_]^3−/4−^ redox couple, were acquired three times to track the principal stages of the assay: (i) the bare electrode, (ii) after sequential adsorption of the thiolated ssDNA probe and MCH, and (iii) after target miRNA adsorption. The probe+MCH modification reduced the peak DPV current relative to the bare electrode, indicating the formation of a partially insulating self‐assembled monolayer (SAM) (Figure 5b). Upon target hybridization, the current decreased further, confirming successful target capture and the formation of a more resistant duplex layer. CV analyses corroborated these observations (Figure 5c), showing diminished anodic/cathodic currents and increased peak‐to‐peak separation (ΔE_p_), reflecting the hindered electron transfer across the functionalized interface. These electrochemical signatures were used to quantify target concentrations, as current attenuation correlated linearly with miRNA concentration, confirming the reliability of hybridization‐based signal transduction.

However, the relatively sharp cathodic feature observed in the CV response may be influenced by the mass‐transport characteristics of the mAu interface. Previous theoretical studies have demonstrated that confinement of electroactive species within porous architectures can introduce thin‐layer diffusion contributions and alter conventional voltammetric behavior, with the extent of these effects depending on parameters such as pore dimensions, scan rate, and diffusion characteristics [37]. Similar pore‐dependent mass‐transport behavior has also been demonstrated experimentally in nanoporous Au, where smaller pores were shown to favor thin‐layer diffusion while larger pores favored bulk diffusion [38]. Therefore, the relatively narrow cathodic feature observed here is plausibly influenced by pore‐confined mass transport within the mAu architecture. The progressive attenuation of the redox peak currents following surface functionalization is also consistent with increasing interfacial blocking and reduced accessibility of the ferri/ferrocyanide redox probe to the Au surface, as previously reported for thiolated nucleic‐acid‐modified Au electrodes [39]. These electrochemical signatures support the hybridization‐based signal‐transduction mechanism.

To further corroborate the interfacial changes observed by CV and DPV, electrochemical impedance spectroscopy (EIS) was performed at the principal stages of sensor fabrication (Figure S6). The bare mAu, probe/MCH‐functionalized mAu, and target‐miRNA‐hybridized mAu electrodes exhibited distinct Nyquist responses in the ferri/ferrocyanide redox electrolyte. Probe/MCH functionalization altered the impedance profile relative to the bare mAu electrode, while a substantially more pronounced change in impedance was observed following hybridization with the complementary target miRNA (Figure S6a), with the impedance response extending toward higher (Z') and (‐Z'') values, particularly at lower frequencies (Figure S6b). This behavior is consistent with increased hindrance of ferri/ferrocyanide‐mediated interfacial electron transfer following formation of the DNA‐RNA duplex, arising from increased steric obstruction and electrostatic effects associated with the negatively charged nucleic‐acid layer. Because fully resolved semicircular regions were not observed within the investigated frequency window, the spectra were interpreted in terms of changes in the interfacial impedance response rather than by direct estimation of charge‐transfer resistance from semicircle diameters.

The analytical selectivity of the proposed miRNA sensing platform was further evaluated using structurally related and non‐complementary RNA sequences. Probe‐functionalized electrodes were challenged with the complementary target hsa‐miR‐200c‐3p and three negative controls: a non‐specific (NS) miRNA, a scrambled (SC) sequence, and a three‐base‐mismatched (MM) sequence. As shown in Figure 5d,e, the complementary target produced a pronounced hybridization‐induced electrochemical response, whereas the NS, SC, and MM sequences generated only minor changes relative to the probe/MCH‐functionalized baseline. The markedly reduced response obtained for the three‐base‐mismatched sequence is particularly important because it demonstrates that the sensing interface can discriminate closely related sequences rather than responding simply to nonspecific RNA adsorption. Similarly, the negligible responses obtained for the scrambled and non‐specific sequences indicate effective suppression of off‐target interactions by the probe/MCH‐functionalized surface. Collectively, these results demonstrate that the electrochemical response is predominantly governed by sequence‐specific hybridization between the immobilized capture probe and the complementary target miRNA, confirming the high analytical selectivity of the sensing platform. To quantify these interfacial changes, the relative adsorption (%iR) was calculated using Equation (1), with the bare‐electrode DPV response used as the common baseline.

(1) $$\%iR\;=\left\{i_{\left\{bare\right\}}-\;i_{\left\{step\right\}}\right\}/\left\{i_{\left\{bare\right\}}\right\}\times 100$$

where *i*_bare_ is the DPV current on the bare electrode, and *i*_step_ is the current after the probe+MCH assembly (I_probe_) or after miRNA hybridization (*i*_miRNA_). We report the %*i*R_probe_ and %*i*R_miRNA_ from the second and third measurements, respectively. The absolute (differential) adsorption attributable to hybridization was computed as %*i*R_a_ = %*i*R_miRNA_ – %*i*R_probe._ As summarized in Figure 5e, %*i*R_probe_ and %*i*R_miRNA_ increased sequentially for the target miRNA as well as the positive control (PC), whereas the negative controls (NS, SC, MM) exhibited negligible blocking, further confirming the selectivity of the assay.

Beyond molecular specificity, the thickness of the mAu electrode can also impact the analytical performance. Therefore, we examined electrodes fabricated with varying numbers of CC (1 to 5), corresponding to progressively thicker films. Although the electrodes with higher CC exhibited larger baseline DPV currents (Figure 4d), the hybridization‐induced response and absolute adsorption (%*i*R_a_) showed the opposite pattern. As shown in Figure 6a, %*i*R_a_ decreased slightly as the number of coating cycles increased, although the trend was not statistically significant (Figure S7; Kruskal‐Wallis test *p* > 0.05). This apparent inverse relationship may arise from thickness‐dependent transport and hybridization kinetics at the electrode interface. A greater number of coating cycles produces a denser, thicker nanostructured layer, which, while enhancing surface area, can also hinder probe immobilization, restrict target access, and increase interfacial resistance. Thicker films may impose greater ion‐diffusion and electron‐transfer impedances, as shown in Figure 4c (inset), reducing the effective signal gain from deeper layers [40, 41]. Thus, the use of a 1 CC electrode with relatively lower material (Figure S1a) without compromising the sensing capability. In addition to the electrode architecture, assay workflow parameters can also influence detection performance. Therefore, we optimized the hybridization incubation time for the target miRNA. As shown in supplementary Figure S8, the hybridization‐induced absolute adsorption increased with time, rising sharply up to 30 min, then modestly to 45 min, and changed only minimally between 45 and 60 min, indicating near‐saturation by 45 min. Accordingly, 45 min was selected as the optimal incubation time for all the subsequent measurements.

**FIGURE 6:**
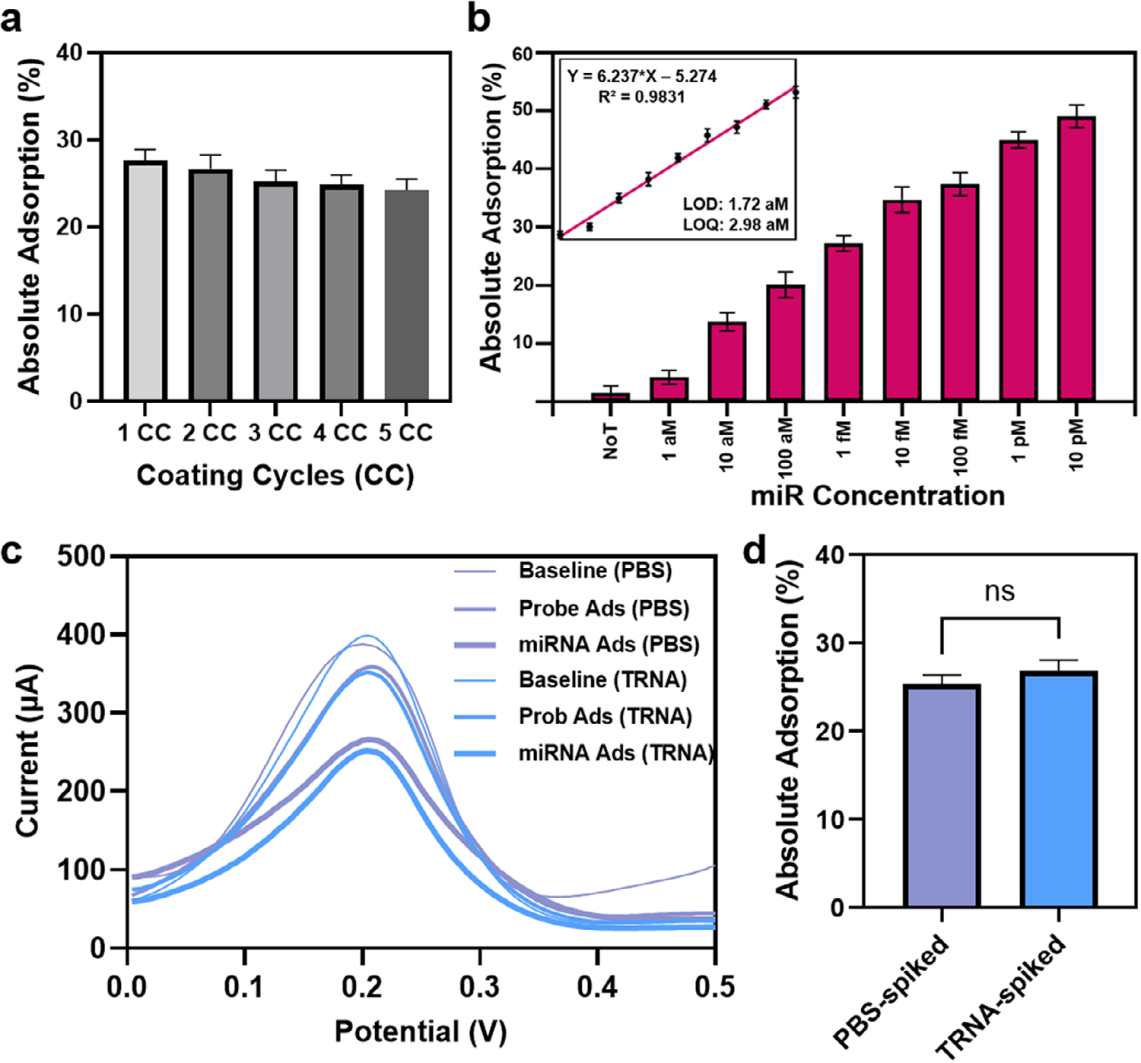
Analytical performance of the mAu‐based miRNA biosensor. (a) Effect of electrode CC (1 to 5) on analytical response. (b) %iR_a_ increased monotonically with hsa‐miR‐200c‐3p concentrations (1 aM‐10 pM). Inset: linear calibration plot (concentration vs. %iR_a_) enabled LOD and LOQ measurements. (c) DPV responses for PBS‐spiked and synthetic total RNA‐spiked samples showed nearly identical peaks. (d) No significant difference was observed between spiked samples on a 1 CC electrode, confirming negligible matrix interference. (Error bars represent mean ± SD from n = 3 independently prepared electrodes).

With optimized incubation conditions in place and having already confirmed negligible specificity crosstalk and only minor effects of coating cycles, we evaluated the platform's sensitivity, dynamic range, and reproducibility. Functionalized mAu electrodes were challenged with synthetic hsa‐miR‐200c‐3p at concentrations ranging from 1 aM to 10 pM using the same assay workflow described above. The quantitative readout was the hybridization‐induced absolute adsorption, expressed as %*i*R_a_ = %*i*R_miRNA_ − %*i*R_probe_, calculated from the DPV peak current and normalized to the bare electrode baseline. As shown in Figure 6b, %*i*R_a_ increased monotonically with the target concentration on a 1 CC mAu electrode. The increase in adsorption corresponded to a decrease in the DPV peak current, consistent with the greater interfacial blocking at higher miRNA levels. The calibration plot of %*i*R_a_ versus concentration showed linearity within the working range (Figure 6b (inset); R^2^ = 0.9831). From the calibration, Y = (6.237 × X) – 5.274, applying signal‐to‐noise ratios of 3 and 10 yielded a limit of detection (LOD) of 1.72 aM and limit of quantification (LOQ) of 2.98 aM, respectively, under the experimental condition used. Replicate measurements across the range yielded an RSD of < 10% (n = 3), indicating good reproducibility. To place our results in perspective, we compared the 1.72 aM LOD of our platform with representative electrochemical miRNA assays reported in the literature. For instance, an electrochemical biosensor employing dual‐cascade isothermal amplification for miR‐21 achieved an LOD of 2.9 aM [42], while an earlier nanostructured electrode system reported an LOD of 5.7 aM for miR‐155 [43]. Therefore, our platform matches or surpasses the sensitivity of many state‐of‐the‐art systems while maintaining a simple, amplification‐free workflow and a short assay time. A broader list of comparable miRNA detection platforms, including their LODs, and assay formats, has been compiled in Supplementary Table S1. This shows that our platform is competitive with state‐of‐the‐art systems, while retaining a simple, amplification‐free workflow. Together with the negligible responses observed for nonspecific, scrambled, and three‐base‐mismatched controls (Figure 5), these results confirm attomolar sensitivity, a broad dynamic range (1 aM to 10 pM), and high reproducibility, underscoring the strong analytical performance of the mAu‐based miRNA biosensor.

Finally, to assess the applicability in a complex matrix, synthetic hsa‐miR‐200c‐3p (100 aM) was spiked into PBS (PBS‐spiked) and, in parallel, into a synthetic total‐RNA solution (TRNA‐spiked). Both samples were processed using an optimized workflow. The resulting DPV profiles were highly comparable with closely aligned peak positions and similar amplitudes (Figure 6c). Quantification of hybridization‐induced absolute adsorption, %iR_a_, showed no statistically significant difference between the matrices (Figure 6d; Mann–Whitney test, p > 0.05). These results indicate negligible matrix interference from background RNA at 100 aM and confirm that the biosensor maintains analytical accuracy and sensitivity in complex nucleic acid environments.

## 3 Conclusions

We present a novel spin‐coating approach for fabricating mesoporous Au thin films on ITO substrates. This method enables facile thickness control, a uniform morphology, and scalable processing. Electrochemical characterization confirmed that the electrochemically active surface area and differential‐pulse voltammetric response increase with film thickness, and biosensing studies demonstrated highly sensitive miRNA detection. Field‐emission SEM and cross‐sectional S/TEM reveal a continuous, crack‐free mesoporous network with pore diameters of ∼17.4 nm and framework widths of ∼21.6 nm at a single coating cycle, and an average film thickness that increases to ∼181 nm per cycle. The interconnected framework is highly crystalline, with lattice spacings of 4.06 Å, 2.34 Å, and 2.8 Å along the [001], [111], and [110] directions, respectively. Electrochemically, the active surface area scales linearly with coating cycles while the charge‐transfer resistance falls to ∼6.4 Ω at six cycles. This study introduces spin coating as a powerful alternative for fabricating mesoporous noble metals and opens new opportunities for bioelectronics and diagnostics. For the sensing demonstration, the working substrate was conductive ITO‐coated glass bearing a thin (∼6 nm) plasma‐induced amorphous ITO surface layer, whereas the spin‐coating method itself is substrate‐agnostic and transferable to insulating and transparent substrates. Building on this platform, the mesoporous architecture offers a large electrochemically active surface area, textured geometry that facilitates redox currents and analyte access, reduced interfacial impedance that supports a high signal‐to‐noise ratio, and high adsorption capacity that concentrates target molecules at the interface. Using these spin‐coated mAu films, we established a simple label‐free miRNA assay that requires no enzymatic or amplification steps, completes within 1.5 h and shows a concentration‐dependent response over the tested range of 1 aM to 10 pM, with a linear calibration relationship (R^2^ = 0.9831) and calculated LOD and LOQ values of 1.72 and 2.98 aM, respectively. Replicate measurements showed RSD < 10%, while nonspecific, scrambled, and three‐base‐mismatched controls produced negligible responses, demonstrating high analytical selectivity.

The present experiments further support short‐term operational retention of the probe‐functionalized interface under the applied electrochemical measurement conditions. However, systematic long‐term storage stability, shelf‐life, regeneration, and repeated‐use performance were not evaluated and remain important considerations for future development. From a translational perspective, the solution‐processable fabrication strategy, controllable film thickness, label‐free and amplification‐free sensing workflow, and compatibility with miniaturized electrochemical readout provide a promising basis for future portable and point‐of‐care diagnostic platforms. Translation toward clinical application will require further assessment of batch‐to‐batch fabrication reproducibility, long‐term storage stability, simplified sample processing and device integration, and validation using clinically relevant biofluids and patient‐derived samples. Overall, the spin‐coated mAu platform provides a versatile and scalable foundation for sensitive, selective, and rapid electrochemical nucleic‐acid detection, with potential extension to additional RNA biomarkers and miniaturized diagnostic devices.

## 4 Experimental Section

### 4.1 Reagents and Materials

All chemicals and solvents were acquired from Sigma Aldrich (USA) and left unpurified unless stated otherwise. Gold (III) chloride trihydrate (HAuCl_4 _·3H_2_O), sulfuric acid (H_2_SO_4_, 98%), and absolute ethanol (Abs. EtOH, C_2_H_6_O), N, N‐dimethylformamide (C_3_H_7_NO, HPLC 99.9%), tetrahydrofuran (THF, C_4_H_8_O, HPLC 99.9%), absolute isopropanol (IPA, C_3_H_8_O, HPLC 99.9%), and sodium citrate (Na_3_C_6_H_5_O_7_). Diblock copolymers with the molecular weight of polystyrene (18 kDa)‐blocked‐polyethylene oxide (7.5 kDa) (PS_18k_‐*b*‐PEO_7.5k_) were obtained from Polymer Source (Canada). All solutions were prepared using double‐distilled deionized water from a Milli‐Q reference water purification system (≥18.2 MΩ·cm at 25°C). Synthetic oligonucleotides (target and non‐complementary miRNAs and thiolated ssDNA probe) were acquired from GenScript (Japan), and sequences are shown in Table S2.

### 4.2 Preparation of mAu Thin Films

The preparation of the mAu thin films is a two‐step process. First, we prepared the mAu ink by mixing 30 mg of PS_18k_‐*b*‐PEO_7.5k_ with 2 mL of a 1:2 ratio of DMF and THF. To this, 1.5 mL of 70 mM HAuCl_4_ (2:1 ratio) and acetylacetone were added dropwise under stirring. Finally, the ink volume was brought to 6 mL by adding deionized water, and the mixture was stirred for 90 min. During this period, Au is reduced, resulting in a change in the ink color (blood red). This can be accelerated by adding 0.1 M sodium citrate. After preparing the spin‐coating ink, ITO glass substrates were treated by exposing the coating side to 60 SCCM oxygen plasma at 120 W for 25 min using an Oxford Plasma Pro 80. Spin coating was performed on the plasma‐treated ITO glass substrates at 600 rpm for 60 s, followed by annealing at 120°C for 60 s. It is to be noted that higher spin speeds can result in the collapse of the soft template micelle, resulting in a non‐porous film, and lowering the spin speeds can result in significant edge‐bleeding as a result of spin coating. The spin‐coating process was repeated until the desired film thickness was reached by increasing the number of CCs. Finally, the sample with the desired CC is set to anneal at 120°C for 6 hrs, under ambient conditions. The spent diblock copolymer was removed by rinsing the electrode in THF at 45°C, followed by N_2_ drying.

Developing this platform required overcoming several practical challenges. Preserving the mesostructure demanded a narrow spin‐coating window, as speeds above 600 rpm collapsed the soft‐template micelles into non‐porous films while lower speeds caused edge‐bleeding. Continuous, crack‐free films were obtained only from three coating cycles onward (Figure S1). High‐power O_2_‐plasma activation of the ITO surface was essential for uniform ink spreading and film adhesion, and template removal by controlled THF treatment and staged annealing was necessary to avoid framework collapse or residual‐carbon contamination. Maintaining stable electrical contact under repeated alligator‐clip connections motivated the use of ITO. Finally, in sensing, thicker films increased surface area but hindered probe immobilization and target access, defining a thickness–accessibility trade‐off that guided the choice of a single‐cycle electrode.

### 4.3 Material and Electrochemical Characterization

The nanostructures of the spin‐coated mAu electrodes were observed using JEOL JSM‐7100F FESEM and imaged at 15 kV, and the chemistry of the mAu electrodes was analyzed through EDX. Detailed cross‐sectional S/TEM images were obtained using a Hitachi HF5000 operated in TEM and STEM configuration on a lamella sample mounted on a Si grid. Crystallographic information for the spin‐coated thin films was recorded using a Rigaku SmartLab XRD instrument with Cu Kα radiation. All electrochemical characterizations were performed using CH Instruments in a 3‐electrode configuration with Ag/AgCl (in 3 M saturated KCl solution) reference and Pt wire as the counter electrode. CV measurements were performed in 0.5 M H_2_SO_4_ over a reduction‐potential range of 1.2 to 0.6 V at scan rates ranging from 10 to 100 mV·s^−1^ to estimate the ECSA. DPV measurements were performed from 0 to 0.4 V in 10 mM (1×) PBS containing 2.5 mM K_3_[Fe(CN)_6_], 2.5 mM K_4_[Fe(CN)_6_], and 0.1 M KCl, using a pulse amplitude of 50 mV and a pulse width of 50 ms. The EIS measurements were performed in pH‐neutral conditions (1× PBS, pH 7.4). However, EIS measurements on thiolated probes were acquired in 1× PBS (pH 7.4) containing 2.5 mM K_3_[Fe(CN)_6_], 2.5 mM K_4_[Fe(CN)_6_], and 0.1 M KCl at a potential of 0.25 V vs. Ag/AgCl. A sinusoidal AC perturbation of 5 mV was applied over a frequency range of 1 Hz to 100 kHz, for bare mAu, probe/MCH‐functionalized mAu, and target‐miRNA‐hybridized mAu electrodes.

### 4.4 Probe Reduction and Formation of a Self‐Assembled Monolayer (SAM)

Thiolated ssDNA capture probes were reduced by adding 2.0 µL of 10 mM TCEP‐HCl (Sigma‐Aldrich, Cat. # C4706) to 20 µL of probe, incubating 45 min at room temperature, and removing excess TCEP with Zeba spin desalting columns (ThermoFisher, Cat. # 89877) per the manufacturer's protocol; TCEP cleaves disulphides to yield free thiols that chemisorb to gold via Au‐S bonds. After baseline measurements on the cleaned electrode, 2.0 µL of the reduced probe (1 µM in PBS) was dispensed on the surface and incubated 15 min in a humid chamber, rinsed 3× with PBS, followed by 2.0 µL 6‐mercapto‐1‐hexanol (MCH; Sigma‐Aldrich, Cat. # 451088) (prepared to give an approximate 50:1 MCH:probe surface ratio) for 15 min to backfill unoccupied Au sites and orient the probe. The electrode was rinsed 3× with PBS and briefly with nuclease‐free water. This procedure forms a mixed probe+MCH SAM that anchors the capture strand and passivates bare gold to suppress nonspecific adsorption; SAM formation was verified by CV/DPV in the [Fe(CN)_6_]^3 − /4 −^ couple (reduced Faradaic current, larger ΔE_p_), yielding the probe‐state current *I*_probe_. Both DPV and CV (scan rates: 50 mV·s^−1^) were recorded in 10 mM PBS (pH 7.4), containing 2.5 mM K_3_[Fe(CN)_6_], 2.5 mM K_4_[Fe(CN)_6_], and 0.1 M KCl solution using an mAu working electrode, Ag/AgCl (in 3 M saturated KCl solution) reference, and Pt counter. DPV was scanned from 0.10 to 0.50 V vs. Ag/AgCl with 50 mV pulse amplitude and 50 ms pulse width. CV was scanned from −0.40 to +0.80 V vs. Ag/AgCl at 50 mV·s^−^^1^.

### 4.5 Electrochemical Detection of miRNA

For hybridization, 30 µL of synthetic hsa‐miR‐200c‐3p (or control strands: nonspecific, scrambled, triple‐base mismatched) prepared in 2× SSC was pipetted onto the probe+MCH‐modified electrode and incubated 45 min at room temperature (humid chamber to prevent evaporation); the electrode was then gently rinsed 3× with PBS (pH 7.4), and DPV/CV were recorded under the same conditions to obtain the post‐hybridization peak current *I*_miRNA_. Using the bare‐electrode current *I*_bare_as a common baseline, relative adsorption (%*iR*) at each state was computed as $\%iR=\frac{I_{\mathrm{bare}}-I_{\mathrm{step}}}{I_{\mathrm{bare}}}\;\times 100;$ for *I*_step_ = *I*_probe_ or *I*_miRNA_, giving %*iR*_probe_and %*iR*_miRNA_, respectively; the hybridization‐induced absolute adsorption was %*i* *R_a_* =  %*iR*_miRNA_ − %*iR*_probe_. All conditions were measured on n = 3 independently prepared electrodes; results are reported as mean ± SD with RSD (%).

## Conflicts of Interest

The authors declare no conflicts of interest.

## Supporting information




**Supporting File 1**: advs77604‐sup‐0001‐SuppMat.docx.

**Supporting File 2**: advs77604‐sup‐0002‐MovieS1.mp4.

**Supporting File 2**: advs77604‐sup‐0003‐MovieS2.mp4.

## Data Availability

The data that support the findings of this study are available on request from the corresponding author. The data are not publicly available due to privacy or ethical restrictions.
