# Supplementary material for: A Scalable Spin‐Coating Strategy for Mesoporous Gold Thin Films toward miRNA Detection
Source: Adv Sci (Weinh). 2026 Oct 8:e77604. Online ahead of print. doi: 10.1002/advs.77604 (PMC13650188; doi:10.1002/advs.77604)
Supplement: Supplementary file 1 [file ADVS-9999-e77604-s003.docx]

Supporting Information

**A Scalable Spin-Coating Strategy for Mesoporous Gold Thin Films towards miRNA Detection**

***Aditya Ashok,^1§^ Emtiaz Ahmed,^1§^ Arya Vasanth,^1^ Mandy Hei Man Leung,^2,3^ Miharu Eguchi,^1,4^ Md Shahriar A Hossain,^1,5^ Mostafa Kamal Masud,^1^* Carlos Salomon,^6^ Yusuke Yamauchi ^1,2,7^****

^1^Australian Institute for Bioengineering and Nanotechnology, The University of Queensland, Brisbane, Queensland, Australia

^2^Department of Materials Process Engineering, Graduate School of Engineering, Nagoya University, Chikusa-ku Nagoya, Japan

^3^Institute for Advanced Research (IAR), Nagoya University, Nagoya, Aichi, Japan

^4^Faculty of Science and Engineering, Waseda University, Shinjuku, Tokyo, Japan

^5^School of Mechanical and Mining Engineering, Faculty of Engineering, Architecture and Information Technology (EAIT), The University of Queensland, Brisbane, QLD, Australia

^6^Translational Extracellular Vesicles in Obstetrics and Gynae-Oncology Group, UQ Centre for Clinical Research (UQCCR), Faculty of Medicine, and UQ Centre for Extracellular Vesicle Nanomedicine, The University of Queensland, Brisbane, Queensland, Australia

^7^Department of Convergent Biotechnology and Advanced Materials Science, Kyung Hee University, 1732 Deogyeong-daero, Giheung-gu, Yongin-si, Gyeonggi-do, South Korea

^§^ These authors have contributed equally to this work.

**Correspondence:** [m.masud@uq.edu.au](mailto:m.masud@uq.edu.au) (M.K.M.); [y.yamauchi@uq.edu.au](mailto:y.yamauchi@uq.edu.au) (Y.Y.)

**Supplementary Figures**

**Figure S1. Surface morphology elucidating uniform surface coverage.** Low magnification images of **(a)** 1 CC (at ×10k mag.), **(b)** 3 CC and **(c)** 5 CC (at ×20k mag.).

**Figure S2. Sample chemistry.** **(a)** EDX spectra on a 3 CC mAu electrode with their respective **(b)** At% in tabular form.

**Figure S3. TEM elemental mapping.** EDX of mAu on ITO/glass **(a)** along the cross-section (scale: 100 nm) and **(b)** at the interface (scale: 50 nm) for (*i*) Au Lα, (*ii*) In Lα, (*iii*) Sn Lα and (*iv*) O Kα, respectively.

**Figure S4. HR-TEM micrographs at the mid-section of the sample.** Lattice-level images of ordered mAu with features **(a)** around a pore structure and **(b)** along the pore edge with Pt redeposition. **(c)** Lattice dislocations along the bulk of mAu film.

**Figure S5. Stability of the probe-functionalised mAu interface under electrochemical interrogations.** FTIR spectra of **(a)** bare mAu and probe/MCH-functionalised mAu electrodes recorded **(b)** before (Pre-CV/DPV) and **(c)** after (Post-CV/DPV) electrochemical measurements. **(d)** Combined FTIR spectra.

***NOTE:*** The probe/MCH-functionalised electrodes display characteristic spectral features within the molecular fingerprint region that are substantially retained following CV/DPV interrogations. The corresponding overlaid spectra demonstrate preservation of the overall spectral profile after application of the electrochemical measurement conditions, supporting retention of the thiolated ssDNA/MCH functional layer on the mAu surface.

**Figure S6. Electrochemical impedance characterisation of the 5 CC mAu sensing interface.** **(a)** Total impedance (Z) as a function of frequency and **(b)** Nyquist plots of bare mAu, probe/MCH-functionalised mAu, and target-miRNA-hybridised mAu electrodes measured using a three-electrode configuration in 2.5 mM ferri/ferrocyanide redox electrolyte, respectively.

***NOTE:*** EIS measurements were performed using a CHI electrochemical workstation at a potential of 0.25 V *vs.* Ag/AgCl with a 5 mV AC perturbation over a frequency range of 1 Hz to 100 kHz.

**Figure S7. Effect of electrode coating cycles on analytical response.** Absolute adsorption (%iR_a_) was measured for electrodes prepared with 1-5 coating cycles (CC). Error bars represent mean ± SD (n = 3). ns = not significant.

***NOTE:*** No statistically significant differences were detected among the coating-cycle groups under the tested conditions (Kruskal–Wallis test, *p* > 0.05).

**Figure S8. Optimisation of miRNA incubation time.** Absolute adsorption measured after 5, 15, 30, 45, and 60 minutes of incubation. The response reaches near saturation by 45 minutes, with only a marginal gain at 60 minutes (error bars = mean ± SD, n = 3).

**Table S1:** Comparison of representative electrochemical miRNA biosensors with the present work.

| **Platform / Strategy** | **Target miRNA** | **Amplification** | **Reported LOD** | **Ref.** |
| --- | --- | --- | --- | --- |
| Mesoporous gold-based electrochemical biosensor | hsa-miR-200c-3p | amplification-free | 1.72 aM | **This work** |
| DNA nanostructures- based electrochemical biosensor | miR-21 | dual-cascade isothermal amplification | 2.9 aM | [1] |
| Screen printed gold electrode-based electrochemical biosensor | miR-155 | dual-signal amplification. | 5.7 aM | [2] |
| Ratiometric electrochemical sensor | miR-21 | amplification-free | 0.15 fM | [3] |
| 3D DNAzyme-walker electrochemical biosensor | miR-155 | DNAzyme walking (amplified) | 56.23 aM | [4] |
| Nanocomposite electrochemical biosensor | miR-155 | Label-assisted (amplified) | 20.2 aM | [5] |
| Homogeneous label-free (G-triplex/MB + λ-Exo) electrochemical biosensor | miR-141 | Enzymatic recycling (λ-Exo) | 16 fM | [6] |

**Table S2:** Synthetic sequences used in this study, including the thiolated ssDNA probe, target miRNA, and non-complementary controls.

| **Type** | **Labelled as** | **Sequence** |
| --- | --- | --- |
| Target miRNA (hsa-miR-200c-3p mature miRNA) | Positive Control/target specific (TS) | 5′- UAA UAC UGC CGG GUA AUG AUG GA-3′ |
| Non-specific miRNA control | Non-specific (NS) | 5′-UCCAGCAUCAGUGAUUUUGUUG-3′ |
| Scrambled negative RNA control | Scrambled  (SC) | 5′-GAUAGCGUACGUUGAGAC CUAUU-3′ |
| Mismatched hsa-miR-200c-3p control | Mismatched (MM) | 5′-UAAGCUUGCCGGGUAAUGAUGGA-3′ |
| Complementary thiolated ssDNA probe | thiolated probe | 5′- TCC ATC ATT ACC CGG CAG TAT TA-/3ThioMC3-D/ -3′ |

**References**

1. Y. Zhao, L. Cheng, M. Xu, and C. Zhao, "Ultra-sensitive detection of microRNAs using an electrochemical biosensor based on a PNA-DNA2 three-way junction nanostructure and dual cascade isothermal amplification," *Analytica Chimica Acta* **1345** (2025): 343755.
2. A. R. Cardoso, F. T. Moreira, R. Fernandes, and M. G. F. Sales, "Novel and simple electrochemical biosensor monitoring attomolar levels of miRNA-155 in breast cancer," *Biosensors and Bioelectronics* **80** (2016): 621-630.
3. H. Shao, X. Xue, Z. Sun, X. Zheng, and P. Shi,  "Detection of microRNA-21 based on smartly designed ratiometric electrochemical sensor and dual-signal amplification." *Analytica Chimica Acta* **1336** (2025): 343444.
4. J. Zhao, C. He, Y. Long, et al., "3D DNAzyme walker based electrochemical biosensor for attomolar level microRNA-155 detection," *Analytica Chimica Acta* **1276** (2023): 341642.
5. F. Khosravi, M. Rahaie, M. R. Ghaani, M. Azimzadeh, and E. Mostafavi, "Ultrasensitive electrochemical miR-155 nanocomposite biosensor based on functionalized/conjugated graphene materials and gold nanostars," *Sensors and Actuators B: Chemical* **375** (2023): 132877.
6. Y. Meng, F. Chen, M. Jiang, et al., "A homogeneous label-free electrochemical microRNA biosensor coupling with G-triplex/methylene blue complex and λ-exonuclease-assisted recycling amplification," *Frontiers in Chemistry* **9** (2021): 753253.
